# Antisera Against Certain Conserved Surface-Exposed Peptides of Nontypeable *Haemophilus influenzae* Are Protective

**DOI:** 10.1371/journal.pone.0136867

**Published:** 2015-09-21

**Authors:** Paul W. Whitby, Thomas W. Seale, Daniel J. Morton, Terrence L. Stull

**Affiliations:** 1 Department of Pediatrics, University of Oklahoma Health Sciences Center, Oklahoma City, Oklahoma, United States of America; 2 Department of Microbiology and Immunology, University of Oklahoma Health Sciences Center, Oklahoma City, Oklahoma, United States of America; Arizona State University, UNITED STATES

## Abstract

Nontypeable *Haemophilus influenzae* (NTHi) cause significant disease, including otitis media in children, exacerbations of chronic obstructive pulmonary disease, and invasive disease in susceptible populations. No vaccine is currently available to prevent NTHi disease. The interactions of NTHi and the human host are primarily mediated by lipooligosaccharide and a complex array of surface-exposed proteins (SEPs) that act as receptors, sensors and secretion systems. We hypothesized that certain SEPs are present in all NTHi strains and that a subset of these may be antibody accessible and represent protective epitopes. Initially we used 15 genomic sequences available in the GenBank database along with an additional 11 genomic sequences generated by ourselves to identify the core set of putative SEPs present in all strains. Using bioinformatics, 56 core SEPs were identified. Molecular modeling generated putative structures of the SEPs from which potential surface exposed regions were defined. Synthetic peptides corresponding to ten of these highly conserved surface-exposed regions were used to raise antisera in rats. These antisera were used to assess passive protection in the infant rat model of invasive NTHi infection. Five of the antisera were protective, thus demonstrating their *in vivo* antibody accessibility. These five peptide regions represent potential targets for peptide vaccine candidates to protect against NTHi infection.

## Introduction

Nontypeable *Haemophilus influenzae* (NTHi) cause both invasive and noninvasive infections, including otitis media, bacteremia and exacerbations of chronic obstructive pulmonary disease [1–4] and are a significant public health burden. The most common infection caused by NTHi is acute otitis media (AOM). AOM accounts for 33% of visits by children to health care centers and is the most frequent reason children receive antibiotics [5]. The incidence of AOM peaks between 6 and 12 months of life; almost 100% of children in developing communities and two-thirds of children in developed communities experience their first episode of OM by one year of age [6]. By age 3 years, 80% of children in the U.S. have experienced at least one episode, and 40% have three or more recurrent episodes [7]. Compared to children without AOM those with acute AOM had 2 additional office visits, 0.2 additional emergency room visits and 1.6 additional prescriptions per year. These visits lead to an estimated incremental increase in outpatient healthcare costs of $314 per year per child [8].

Historically, *Streptococcus pneumoniae* were the most common AOM isolate, and NTHi were the second most common [5]. Since the introduction of the PCV-7 *S*. *pneumoniae* vaccine in 2000, the number of cases of OM attributable to *S*. *pneumoniae* has markedly decreased [5,9]. However, the overall number of cases of OM has been reduced about 7% with the PCV-7 vaccine [5,10]. The reduction in the incidence of *S*. *pneumomiae* OM has resulted in an increase in the proportion of OM attributable to NTHi, and NTHi is now reported as the predominant cause of AOM [5,11,12].

In previous decades, greater than 95% of the cases of invasive disease caused by *H*. *influenzae* were due to strains with the type b capsule. However, vaccines based on the type b capsular polysaccharide have virtually eliminated such infections in regions where the vaccine is extensively used [13]. Nevertheless, NTHi strains continue to cause invasive disease principally in perinatal infants, young children, and those older than 65 years [1,14].

Several lines of evidence suggest that prevention of AOM due to NTHi is possible [15–17]. First, AOM is largely a disease of infants in whom the serum and mucosal antibodies directed against common pathogens are low [18]. Second, OM-prone children have lower levels of serum antibodies than healthy age-matched controls [19,20]. Third, individuals with immunodeficiencies are predisposed to repeated NTHi infections [21]. In addition, breast-feeding is associated both with a reduced frequency of AOM, and higher levels of serum antibodies against NTHi in the nursing infant [22].

Evidence from animal studies also supports the possibility of preventing AOM caused by NTHi. For example, it is possible to protect against challenge by pre-immunization with pilins from the challenge isolate, although cross protection against unrelated isolates was not developed [23]. Similarly, peptide motifs of the pilins were shown to protect, but only against homologous challenge [24]. This lack of cross protection presumably results from known sequence heterogeneity of the pilin proteins. Other studies have assessed protection afforded by antibodies to a number of virulence factors, including major and minor outer-membrane proteins (OMPs) and lipooligosaccharide [17]. Finally, an 11-valent *S*. *pneumoniae* vaccine using *H*. *influenzae* protein D as a carrier molecule afforded partial protection (a reduction of 35%) against NTHi OM in a human clinical trial [16,25,26].

Non-toxic, broadly cross-reactive immunoprotective NTHi antigens have yet to be identified, and there are significant obstacles to be addressed before a comprehensive vaccine can be developed for NTHi. Because of the known heterogeneity of surface associated moieties in the NTHi [27], an efficacious vaccine may require a multi-component design. While a few vaccine candidates have been identified, the development, production and clinical testing of vaccines against NTHi are still lacking [17]. To date, highly immunogenic surface components that elicit protective antibodies against mucosal infections by all strains of NTHi in the age-appropriate population have not been identified. As a first step to identifying suitable protective epitopes, we used a genomic analysis to identify conserved surface-exposed peptides and tested 10 of these for their *in vivo* antibody accessibility.

## Materials and Methods

### Bacterial strains and growth conditions

The NTHi strain R2866 was isolated from the blood of an immunocompetent child with clinical signs of meningitis subsequent to AOM [28]. We have previously utilized this strain in the infant rat model of invasive *H*. *influenzae* disease [29].

NTHi strain sequences used to generate alignments included sequences available through GenBank as well as multiple strains sequenced in house. Sequences obtained through GenBank were from the following strains: 3655, 6P18H1, 7P49H1, PittAA, PittEE, PittGG, PittHH, PittII, R3021, R2846, R2866, 22.1–21, 22.4–21, 86-028NP and NT127. Strains sequenced in house were from our laboratory collection and included several selected from those typed by electrophoretic mobility of 15 metabolic enzymes [30]. These strains were selected to represent the breadth of the species as defined by electrophoretic type (ET) and were HI1373, HI1374, HI1388, HI1394, HI1408, HI1417 and HI1426 representing, respectively, ET’s 13, 26, 43, 53, 68, 77 and 86. An additional four clinical isolates selected from our collection were also sequenced: HI1722, HI1974, HI2114 and HI2116. Isolates of *H*. *influenzae* were routinely maintained on chocolate agar with bacitracin at 37°C. Broth cultures of *H*. *influenzae* were grown in brain heart infusion (BHI) agar supplemented with 10 μg/ml heme and 10 μg/ml β-NAD (supplemented BHI; sBHI).

### Genome sequencing of NTHi strains

Chromosomal DNA was isolated from bacteria recovered from fresh 12 hour broth cultures using the DNeasy Blood and Tissue Kit (Qiagen) as described by the manufacturer. Genome sequences of the NTHi strains were determined at the Laboratory for Molecular Biology and Cytometry Research, University of Oklahoma Health Sciences Center.

NTHi samples were prepared using 50-ng of total genomic DNA according to Nextera DNA library kit protocols (Illumina Inc). Samples were indexed according to standard protocols so that they could be pooled together and sequenced simultaneously in a single run on the Illumina MiSeq using paired-end 150-bp (300 cycle) chemistry. Prior to sequencing, all libraries were run individually on the Agilent High Sensitivity DNA chip to confirm library quality and average insert size. Samples were pooled in equimolar amounts and 8pM of the pool was run on the sequencer. Per Illumina’s recommendation, phiX control was spiked into the library pool prior to loading for quality control purposes (5–10% phiX). Thirty to forty million reads total were collected for each run. Raw sequence data were aligned to a reference isolate using CLC Genomics Workbench (CLC Bio.) to identify single-nucleotide polymorphisms (SNPs) as well as regions with insertions or deletions (indels) or raw data files were assembled using CLC *de novo* assembly parameters.

The genome sequences generated herein were deposited in Genbank. Sequenced strains and corresponding accession numbers are as follows HI1373 (LFDP00000000), HI1374 (LFDO00000000), HI1388 (LFDN00000000), HI1394 (LFDM00000000), HI1408 (LFDJ00000000), HI1417 (LFDK00000000), HI1426 (LFDL00000000), HI1722 (LFFU00000000), HI1974 (LFFT00000000), HI2114 (LFFR00000000), and HI2116 (LFFS00000000).

### Identification of SEPs present in all NTHi strains

Initially, the complement of putative surface-exposed proteins (SEPs) of the isolate NTHi 86-028NP was determined based on the reported annotation of this isolate [31]. For the purposes of this paper SEPs include characterized and putative OMPs as well as other secreted proteins. Hypothetical proteins were individually examined to determine the presence of leader sequences and/or other indicators that they may be secreted or membrane bound. All identified proteins were then used to query the presence of homologs in the other sequenced NTHi. Absence of a homolog in any of the sequenced NTHi excluded that protein from further consideration. Once each core SEP was identified, Geneious software was used to perform sequence alignments with all the known homologs of a given protein in all the available NTHi genomes.

### Molecular modeling

The identified core SEP genes of NTHI 86-028NP were individually examined to determine homology to other known structurally defined proteins. Structures were generated using the Modeller algorithm [32,33], Swissmodel [34] and Phyre2 [35] via The Protein Model Portal [36]. Putative structures were compared and visualized using Chimera to determine potentially surface-exposed regions [37]. Proteins that shared no significant similarities with other modeled proteins were examined to determine regions indicative of secondary structure using PRED-TMBB, BOMP (β-barrel), and TMHMM (α-helix) [38–40].

### Selection of peptides

From these models, predicted surface-exposed regions greater than 10 amino acids long were selected. Multiple sequence alignments were performed with each core protein. All NTHi homologs of each protein from both complete and partial gene and genome sequences were used to perform these alignments. For the majority of proteins, more than 40 NTHi sequences were aligned. External regions greater than 10 amino acids in length were further examined to identify the degree of conservation of sequence across the NTHi. Regions with high conservation were selected as potential antigens. Some selected external loops were longer than 25 amino acids. In these cases AbDesigner [41], was used to determine the most immunogenic region. A truncated synthetic peptide was then selected from this region for further study. Synthetic peptides with >95% purity were synthesized by SynBioSci Corp., Peptide 2.0 Inc., or Thermo Fisher. During synthesis, an aliquot of each peptide was conjugated to Keyhole limpet haemocyanin (KLH) to facilitate immunization studies. A second aliquot was conjugated to biotin for use in ELISA assays.

### Immunization of rats

Antisera against each synthetic peptide were raised either in-house or by Thermo Fisher in two adult Sprague-Dawley rats (~300g) using an 80 day protocol. Initially a pre-immune bleed of approximately 1 ml was performed on each rat. On the following day rats were immunized with 100 μg of antigen in Complete Freund’s Adjuvant. Booster injections were performed on days 21, 42 and 62 with 50 μg of emulsified peptide preparation in Incomplete Freund’s Adjuvant. All immunizations were administered subcutaneously to the dorsum at four to six separate locations to minimize swelling and distress. On day 50, serum samples were collected and antibody titers determined by peptide specific ELISA. Samples with a titer in excess of 3200 were considered suitable for protection studies and these animals were exsanguinated on day 80. Antisera from Thermo Fisher were shipped on dry ice. All antisera were stored, frozen at -80°C until protection studies were performed.

### Rat model of NTHi bacteremia

The rat model of bacteremia following intraperitoneal infection with *H*. *influenzae* was used to compare the abilities of antisera to protect against invasive disease as previously described [42–44]. Specified pathogen free (SPF), timed-pregnant Sprague-Dawley rats (Charles Rivers) were received approximately five days prior to giving birth. These pregnant females were single housed on hardwood litter with *ad libitum* access to water and a standard pelleted food (Purina Lab Rodent Diet 5001). They were maintained on a 12 hour light-dark cycle in separate forced air cubicles in a bio-containment facility (ABSL2) to prevent cross-contamination. Newborn pups from different mothers were pooled and randomly reassigned to the mothers (n = 8–10 pups per female).

In each experiment, cohorts of 4-day old infant rats were injected subcutaneously with 100 μl of either pre-immune serum, antiserum raised to a specific peptide, or PBS. The following day each infant rat was challenged by intraperitoneal injection of approximately 1.5x 10^5^ CFU of R2866. Inocula were prepared as previously described [44], and the actual infective dosage was confirmed by quantitative plating. At 24 or 48 hours post-infection, blood samples (50 μl) were obtained from the anesthetized infant rats (gaseous isoflourane) by cardiac puncture. Bacterial titers were determined using a modified track-dilution method as previously described [44]. All plates were incubated at 37°C for 24–48 hours to quantify CFU/ml. The Fisher Exact Test was used to determine the statistical significance of differences in the fraction of animals developing bacteremia in different infant rat cohorts. The Kruskal-Wallis test was used to determine the statistical significance of differences in the mean bacteremic titers between groups of infant rats. A *P* value <0.05 was taken as statistically significant.

### Ethics statement

This study was performed in strict accordance with the recommendations in the Guide for the Care and Use of Laboratory Animals of the National Institutes of Health. The protocols for use of animals in this study were reviewed and approved by the Institutional Animal Care and Use Committee of the University of Oklahoma Health Sciences Center (Protocol numbers 13-139-H and 14-029-I). Infected animals were monitored for signs of pain and stress at least twice daily following infection. Signs of pain and stress are gasping, writhing behavior, immobility, vocalization, hypothermia and abnormal color and were criteria for the affected animal to be immediately euthanized. No animals were euthanized due to signs of pain or stress in the study. All animals were euthanized by carbon dioxide inhalation following the blood draw.

## Results

### Immunological examination of HxuC peptides

Preliminary experiments were performed to gauge the likelihood that conserved surface-exposed peptides represent protective epitopes. These experiments were initiated when the available sequenced *H*. *influenzae* genomes were limited. Five complete genomes were utilized in these studies and were the originally sequenced isolate Rd KW20, a sequenced type b isolate 10810, and three NTHi isolates (86-028NP, R2866 and R2846). The protein of interest in these studies was the heme-hemopexin utilization protein HxuC [45,46]. The HxuC protein sequences from the five genome-sequenced isolates, as well as several stand-alone HxuC protein sequences from additional strains, were used to perform sequence alignments. At the same time a predicted molecular model was constructed and the putative surface-exposed regions were determined. Five regions showing a high degree of sequence conservation were selected for immunological examination (Table 1).

**Table 1:** Peptide Sequences Selected for Polyclonal Antisera Production.

Protein^a^	Peptide Sequence^b^
HxuC1	LYNNKTIEKEQRKV
HxuC2	DHYDTSSKTVKYKD
HxuC3	APSMQERFVSGAHFG
HxuC4	KGKDKDSGEALSNIAASK
HxuC5	ENLFDRKYQPAFSLMEGTGRN
ComE1	TLNKDDG(V/I)YYLNGSQSGKGQ
Hel1	DNSPYAGWQVQNNKPFDGKD
Hel2	GDNLDDFGN(T/S)VYGKLNADRR
TdeA1	QRRVDISTNSA(I/T)SHK
OmpU1	SWDYQKSTSNHAFYRYDKNR

^a^ Annotated name of the protein in the NTHi isolates (suffix indicates peptide number).

^b^ Amino acid sequence of the select peptide. Residues in parentheses represent variant residues at that single position.

**Fig 1:**
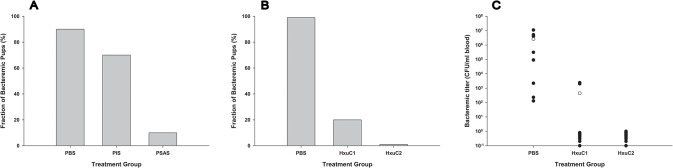
Protection afforded by anti-HxuC antisera in the infant rat model of NTHi bacteremia. Panel A) Percentage of infected infant rats pretreated with pentavalent anti-HxuC antisera with detectable bacteremia 48 hours after infection. Twenty-four hours prior to infection cohorts of 10 infant rats were pretreated with phosphate-buffered saline (PBS), pre-immune serum (PIS) or peptide-specific antiserum (PSAS). Using Fisher’s exact test to compare percentages of bacteremic pups *P* = 0.0011 for PBS vs PSAS and *P* = 0.0198 for PIS vs PSAS. Panel B) Percentage of infected infant rats pretreated with antisera against specific HxuC peptides with detectable bacteremia 48 hours after infection. Twenty-four hours prior to infection cohorts of infant rats were pretreated with phosphate- buffered saline (PBS; cohort of 8 rats), HxuC1 specific antisera (HxuC1; cohort of 10 rats), or HxuC2 specific antisera (HxuC2; cohort of 10 rats). Using Fisher’s exact test to compare percentages of bacteremic pups *P* = 0.0011 for PBS vs HxuC1 and *P* = 0.0001 for PBS vs HxuC2. Panel C) Bacteremic titers in infected infant rats pretreated with antisera against specific HxuC peptides 48 hours after infection. Filled dots represent the bacteremic titer in each individual animal in a cohort. The unfilled dot represents the average bacteremic titers in all members of the cohort. Values of 1 or below represent animals with no detectable bacteremia. Using the Kruskal-Wallis test to compare bacteremic titers(means ±SD) *P* = 0.002 for PBS vs HxuC1 and *P* = 0.0004 for PBS vs HxuC2.

In a screening experiment the five KLH-conjugated peptides were mixed to provide a pentavalent preparation that was used to immunize two adult rats as described. Antisera raised to this pentavalent peptide preparation provided significant protection against NTHi bacteremia by comparison with both PBS control and pre-immune sera from the same animals (Fig 1A).

Having demonstrated that antisera raised to the pentavalent-peptide preparation were protective we examined each of the five HxuC-derived peptides individually. Antisera specifically against two of the peptides (HxuC1 and HxuC2) were highly protective (Fig 1). All animals receiving antisera to HxuC2 failed to develop bacteremia, while in the cohort receiving HxuC1 antisera 2 of 10 infected animals developed bacteremia (Fig 1B). In the two animals in the HxuC1-antisera treated group that developed bacteremia, the bacterial titers were approximately 1000-fold less than control animals (Fig 1C). Antisera to the remaining three peptides from HxuC did not provide statistically significant protection against NTHi invasive disease.

Since certain peptides derived from HxuC gave rise to protective antisera we extended our study to include additional potentially surface-exposed proteins from *H*. *influenzae*. Since a highly effective vaccine is available to prevent type b disease, and type d strains are essentially avirulent we excluded strains 10810 and Rd KW20, and instead focused on the more clinically relevant NTHi strains.

### Genome sequencing of genetically characterized diverse NTHi isolates

**Fig 2:**
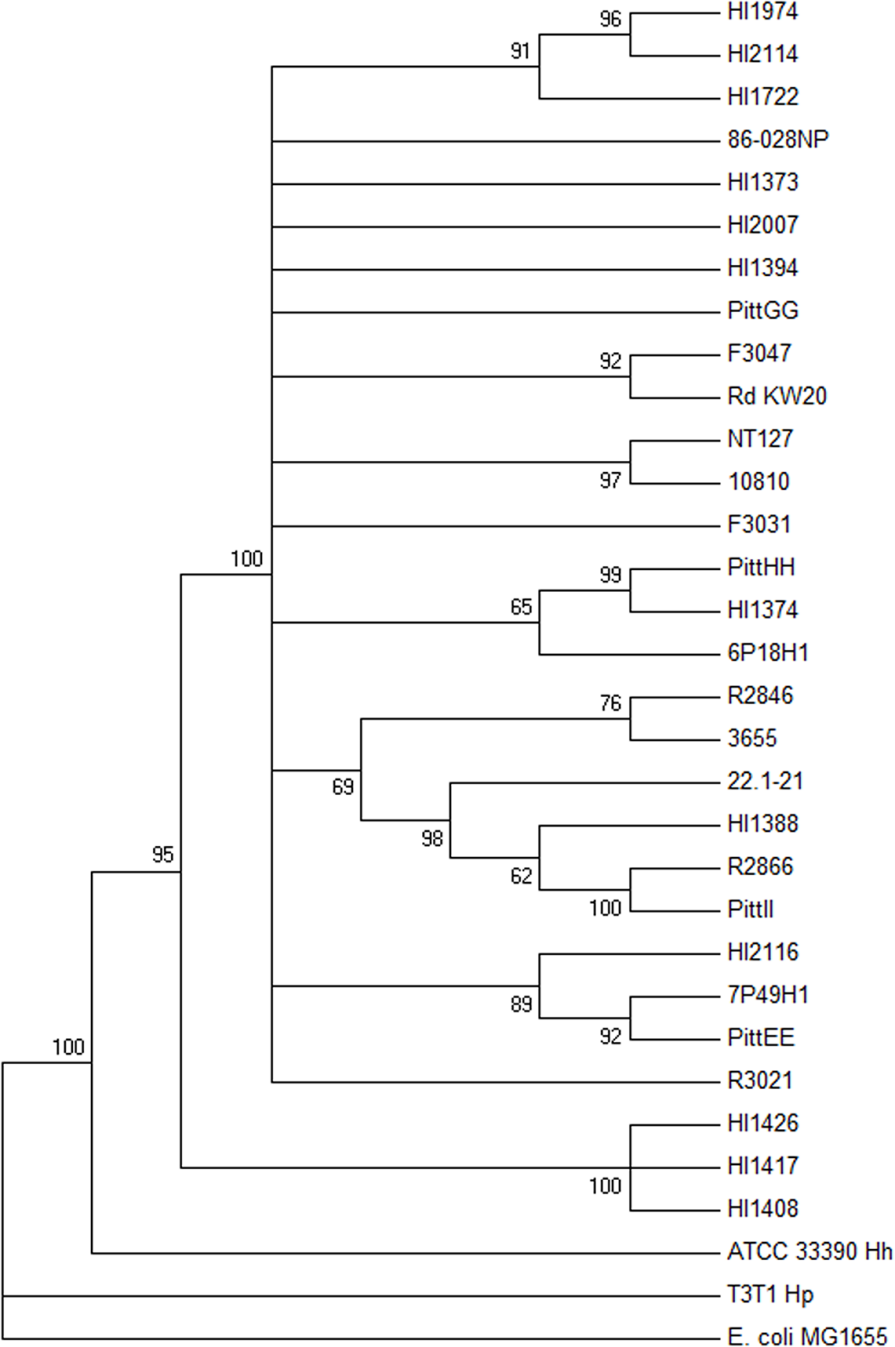
Distribution of sequenced NTHi isolates. Neighbor joining dendogram of NTHi strains used in this study. The tree is rooted with *Eschericia coli* MG1655 and is based on sequence comparisons of the concatenated *adk*, *pgi*, *recA*, *infB* and 16s rRNA gene sequences, with bootstrap values of greater than 50% of 1,000 bootstraps indicated. Also included are several non NTHi sequences; Hp (*H*. *parainfluenzae* T3T1), HH (*H*. *haemolyticus* ATCC 33390) and the *H*. *influenzae* strains Rd KW20 and 10810.

At the time that the HxuC peptides were selected, the number of sequenced genomes was too low to confidently determine conservation across the NTHi of any single gene and insufficient to determine the breadth of variation of each individual surface-exposed loop. Currently over 30 NTHi genome sequences are publicly available,owever, only nine of these sequences are complete. The rest are only useful to confirm the presence and sequence of a particular gene within the respective genome. However, since all genes may not be present, absence of an SEP in an inadequately sequenced genome cannot exclude it from further consideration as a core SEP. To enhance the probability that the peptide selection included regions found in all NTHi, the genomes of an additional 11 NTHi isolates were sequenced. To assure genetic diversity, isolates for sequencing were chosen from strains previously used to define the breadth of the species by electrophoretic typing [30], as well as other NTHi clinical isolates from our culture collection. A multi locus sequence analysis system based on five gene loci (*adk*, *pgi*, *recA*, *infB*, and 16S rRNA) was applied to these newly sequenced genomes [47]. Using these concatenated sequences from all the sequenced NTHi a dendrogram was constructed to demonstrate the distribution of the newly sequenced isolates within the species (Fig 2).

### Identification of core SEPs present in the NTHi

Initially we identified the complement of putative SEPs in the NTHi strain 86-028NP. Such proteins were identified based on known annotation, the presence of export signal sequences, and their similarity to known OMPs in other species. Proteins that localize exclusively to the periplasmic space were in general excluded. However, some proteins considered periplasmic have been shown to be also externally expressed; for example Pal has recently been shown to be inserted in the OM in two distinct orientations [48,49]. Thus, periplasmic proteins that might be anchored to the OM were retained unless definitive evidence exists that they are not externally located. Each coding region was analyzed using PSORTb and PSORT [50]. Proteins with localization signals indicating export across the cytoplasmic membrane were analyzed for homology to experimentally determined OMPs from other organisms. Finally, those proteins in which localization to the OM was putative were further subjected to analysis for structural motifs indicative of membrane-spanning domains. Ninety-six SEPs were identified in strain 86-028NP. This data set was then used to establish the presence of each allele in each of the 21 complete NTHi genome sequences. From these 21 complete sequences, a set of 56 NTHi core SEPS was identified (Table 2). Using all of the available genome and stand-alone gene sequences, the sequence conservation of each individual OMP gene was determined.

**Table 2:** Core SEPs of the NTHi^a^.

86-026NP locus	Gene designation	Rd KW20 locus	Gene description	Probable Type^b^
NTHI0579	*ytfL*	HI0452	Putative hemolysin (probable inner membrane)	α-helix
NTHI0576		HI0449	Conserved hypothetical protein	Amorphous
NTHI0560	*comE*	HI0435	Outer membrane secretin ComE	**Amorphous**
NTHI0522	*ompP1*	HI0401	Outer membrane protein P1	**β-barrel**
NTHI0509	*yeaY*	HI0389	Slp family OM lipoprotein	Amorphous
NTHI0501	*pal*	HI0381	Peptidoglycan associated OMP	**Amorphous**
NTHI0486	*pilF*	HI0366	Transformation and Tfp-related protein PilF	**Amorphous**
NTHI0449	*oapB*	HI0331	Opacity associated adhesion protein B	**Amorphous**
NTHI0448	*oapA*	HI0330	Opacity associated adhesion protein A	**α-helix**
NTHI0409	*pilA*	HI0299	Type II secretory pathway, major prepilin PilA	**Amorphous**
NTHI0370	*hxuB*	HI0263	Heme-hemopexin utilization protein B	**β-barrel**
NTHI0369	*hxuC*	HI0262	Heme-hemopexin utilization protein C	**β-barrel**
NTHI0363	*nlpB*	HI0256	OMP assembly complex subunit NlpB/BamC	**Amorphous**
NTHI0354	*hap*	HI0247	Adhesion and penetration protein precursor	**β-barrel**
NTHI0353		HI0246	Putative lipoprotein	**Amorphous**
NTHI0338	*mltF*	HI0232	Membrane-bound lytic murein transglycosylase F	**Amorphous**
NTHI0303	*nucA*	HI0206	5’-nucleotidase NucA	**Amorphous**
NTHI0267	*ompE*	HI0178	Adhesin protein E	**Amorphous**
NTHI0266	*bamD*	HI0177	OMP assembly complex subunit BamD	**Amorphous**
NTHI0252	*yajG*	HI0162	Putative lipoprotein	**Amorphous**
NTHI0225	*ompP2*	HI0139	Outermembrane protein P2	**β-barrel**
NTHI0220		HI0134	Putative OMP assembly protein	β-barrel
NTHI0205	*mltA*	HI0117	Membrane-bound lytic murein transglycosylase A	**Amorphous**
NTHI0202	*hemR*	HI0113	Probable TonB-dependent heme receptor	β-barrel
NTHI1987	*yccT*	HI1681	Conserved hypothetical protein	**Amorphous**
NTHI1957	*lppC*	HI1655	Lipoprotein LppC	**Amorphous**
NTHI1954	*spr*	HI1652	Lipoprotein Spr, probable murein endopeptidase	**Amorphous**
NTHI1930		HI1236m	Conserved hypothetical protein	**β-barrel**
NTHI1668	*tdeA*	HI1462	Outer membrane efflux porin TdeA	**β-barrel**
NTHI1794m		HI1369	Probable TonB-dependent transporter	β-barrel
NTHI1473	*lpp*	HI1579	15 kDa peptidoglycan-associated lipoprotein	**α-helix**
NTHI1435	*lolB*	HI1607	OM lipoprotein insertion protein LolB	**Amorphous**
NTHI1390	*hup*	HI1217	Heme utilization protein	β-barrel
NTHI1387		HI1215	Conserved hypothetical protein	**Amorphous**
NTHI1342	*olpA*	HI1174m	Probable surface adhesion OlpA	**β-barrel**
NTHI1332	*ompP5*	HI1164	Outer membrane protein OmpP5	β-barrel
NTHI1262		HI1098m	Conserved hypothetical protein	Amorphous
NTHI1171	*ompU*	HI0997m	Putative OM protein OmpU	**β-barrel**
NTHI1169	*tbp2*	HI0995	Transferrin binding protein 2	**Amorphous**
NTHI1168	*tbp1*	HI0994	Transferrin binding protein 1	β-barrel
NTHI1164	*igA1*	HI0990	IgA1 protease	**β-barrel**
NTHI1140		HI0966	Conserved hypothetical protein	**β-barrel**
NTHI1133	*ycfL*	HI0960	Putative lipoprotein YcfL	**Amorphous**
NTHI1101		HI0930	Putative lipoprotein	Amorphous
NTHI1091	*lptE*	HI0922	LPS assembly OM complex LptDE component	**β-barrel**
NTHI1084	*bamA*	HI0917	OM protein assembly factor BamA	**β-barrel**
NTHI1005	*smpA*	HI0838	OMP assembly complex subunit SmpA/BamE	**Amorphous**
NTHI0921	*mltC*	HI0761	Membrane bound-lytic murein transglycosylase C	**Amorphous**
NTHI0889	*lptD*	HI0730	LPS assembly OM complex LptDE, protein LptD	**β-barrel**
NTHI0849	*mlaA*	HI0718	Outer membrane lipid asymmetry protein MlaA	α-helix
NTHI0840m	*hgpC*	HI0712	Hemoglobin-haptoglobin utilization protein C	β-barrel
NTHI0821	*tpsA*	HI0698	Two-partner secretion system protein	**β-barrel**
NTHI0820	*tpsB*	HI0696	Two-partner secretion system protein	**β-barrel**
NTHI0816	*hel*	HI0693	Outer membrane protein P4	**Amorphous**
NTHI0811	*glpQ*	HI0689	Glycerophosphodiesterase	Amorphous
NTHI0782	*hgpB*	HI0661	Hemoglobin-haptoglobin utilization protein B	β-barrel

^a^ Proteins were initially identified as putative members of the SEP complement using PSORT and PSORTb analysis of cellular localization of predicted protein sequences and/or due to homology to known OM localized proteins. Lists were narrowed by excluding SEPs not conserved across the sequenced NTHi isolates and removal of proteins that lacked a strong probability of being localized to the outer membrane and having surface exposed residues.

^b^ Probable structure based on modeling. PRED-TMBB and BOMP (β-barrel), TMHMM (α-helix), amorphous for proteins that fit neither model or have components of both. Bolding indicates proteins that have been modelled and for which conserved peptideshave been identified (see S1 Table).

### Molecular modeling to assess surface-exposed regions of the SEPs

**Fig 3:**
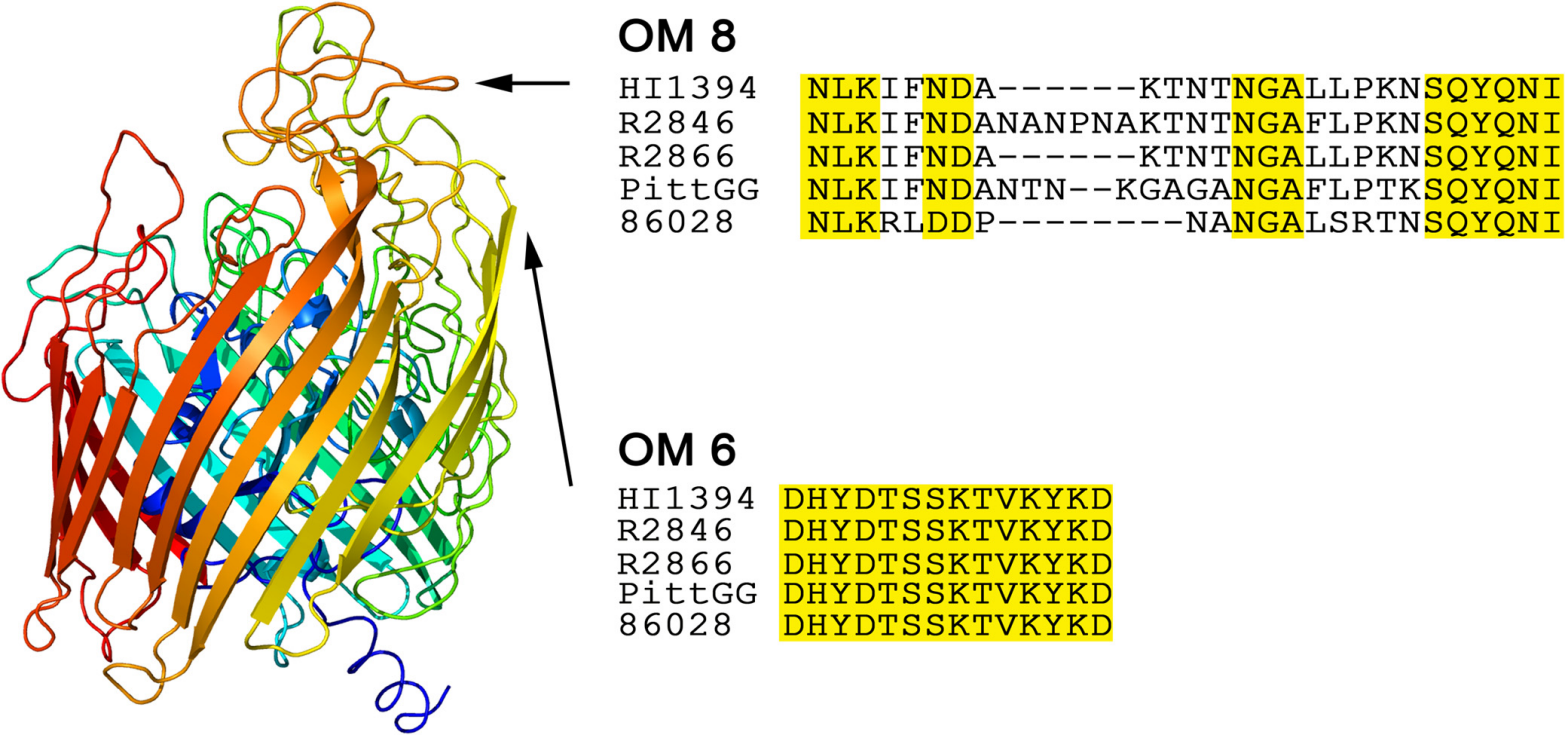
Outer membrane loops of an NTHi SEP. 3D computer-predicted spatial topography of the gated TonB-dependent porin HxuC. The ribbon area corresponds to the external membrane embedded barrel. Also shownare surface exposed external loops. Arrows point to two specific loops designated OM loops 6 and 8. Alignments of both loop regions from five NTHi strains are shown. OM loop 8 is highly heterologous while OM loop 6 is conserved.

The core SEPs s fall into three main structural categories, β-barrel, α-helix and amorphous. The majority of OMPs that are embedded in the membrane adopt the β -barrel structure while the remaining have an α-helix based structure. The OMPs that are either secreted or bound to the outer membrane by a small lipophilic tail are more amorphous, often with no clearly defined common structural features. Our previous studies focused on HxuC, a defined OMP with the β -barrel conformation. In the outer membrane, such proteins fold to create a barrel-like structure with a core, or plug, which can be shifted to allow ingress of a transported molecule (Fig 3) [51,52]. Referred to as “gated porins”, these OMPs have been the focus of numerous X-ray crystallization studies. Since they are structurally constrained, it is possible to both map the NTHi OMPs to those with known crystal structure and to use computer assisted molecular modeling algorithms to determine the potential externally-exposed loops. In some cases an external loop is small, comprising one or two residues while other loops are longer and show variable degrees of sequence heterogeneity. The proposed structure of HxuC is shown to demonstrate the topography and location of the OM loops (Fig 3). Adjacent to it are two sequence alignments that correspond to the indicated loops (for simplicity, only 5 genome sequences are included). Loop 8 is highly heterologous and thus does not constitute a good candidate for further study (Fig 3). In contrast loop 6 is conserved and satisfies our criteria for selection as a suitable peptide motif for generation of antisera. Loop 6 corresponds to the protective peptide HxuC2 described above. Similarly, the OMPs determined to have the α-helix conformation were mapped where possible to the conserved residues of OMPs in other species that have deduced crystal structures. OMPs which are loosely attached to the membrane have proven more difficult to map. To determine potentially exposed regions on these OMPs, we utilized numerous molecular prediction algorithms to identify potential transmembrane and exposed residues. These are usually based on hydrophobicity/hydrophilicity plots and periodicity of residues in these regions. Such analyses can be used to potentially identify regions of interest without providing an elaborated structure as shown for NTHI1734m (Fig 3). Of the 56 core OMPs, 24 appear to have the β-barrel structure and four the α-helix structure while the rest are amorphous structures anchored to the membrane by a polypeptide tail. To date, 41 of the core OMPs have been sufficiently modeled to identify surface exposed peptide motifs. These include 16 of the β-barrels, 2 of the α-helical proteins and 23 of the amorphous structures (Table 2). Combining putative structure with the sequence alignments allows identification of conserved putatively surface exposed regions (S1 Table and S2 Table).

### Characterization of protective epitopes

Although the structural mapping indicates conserved surface exposed regions, determination of whether these regions are antibody accessible *in vivo* requires empirical analysis. From the sequence alignments, 5 external OM loops that showed conservation and that were a minimum of 10 amino acid residues in length were selected. The 5 selected epitopes were in addition to the 5 from HxuC peptides examined above. The 5 new epitopes were from 4 different proteins ComE, Hel, TdeA and OmpU and were designated respectively ComE1, Hel1, Hel2, TdeA1 and OmpU1 (Table 2)

**Fig 4:**
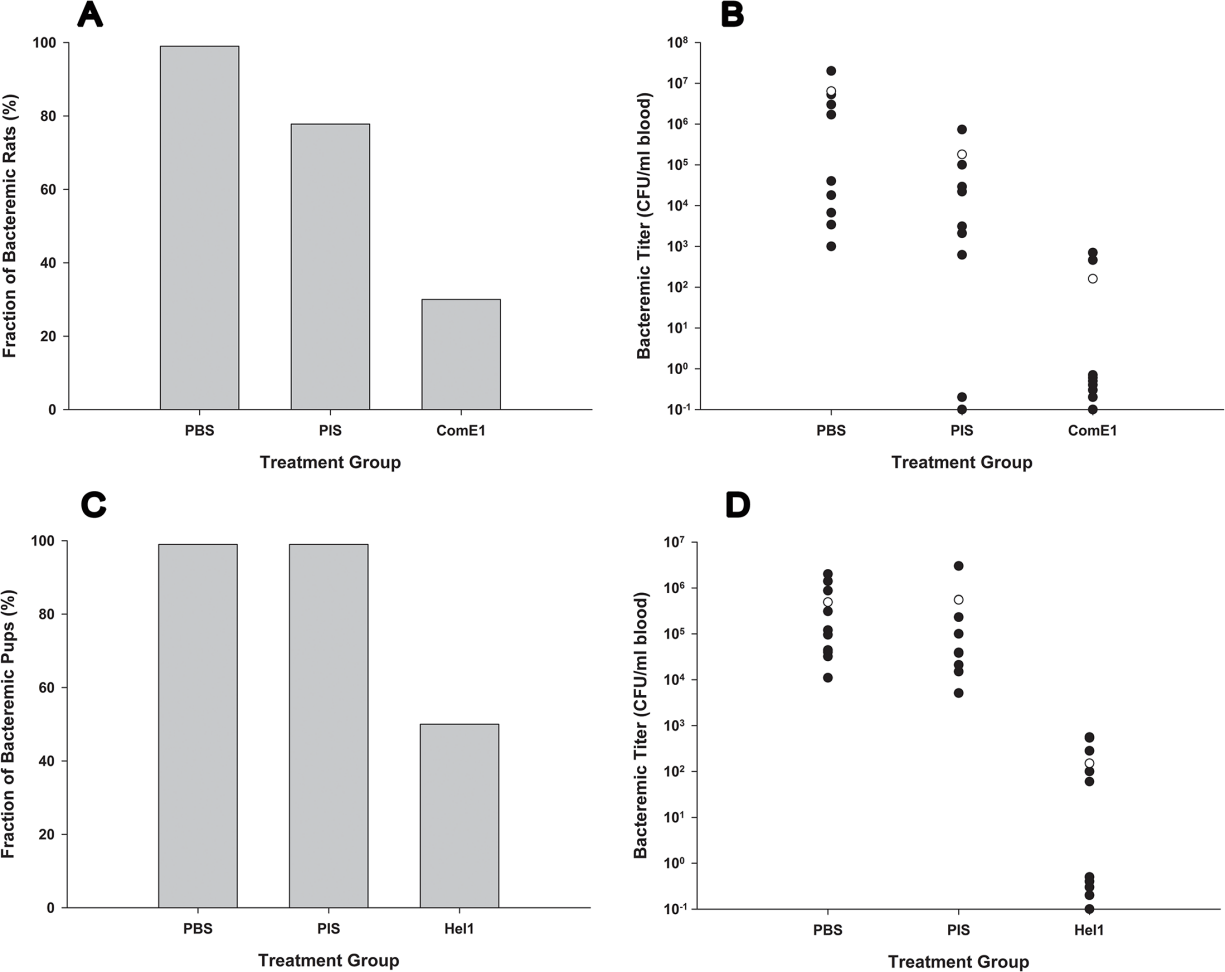
Protection afforded by antisera raised against ComE and Hel derived peptides in the infant rat model of NTHi bacteremia. Panel A) Percentage of infected infant rats pretreated with anti-ComE1 antiserum with detectable bacteremia 24 hours after infection. Twenty-four hours prior to infection cohorts of infant rats were pretreated with phosphate-buffered saline (PBS; cohort of 9 rats), pre-immune serum (PIS; cohort of 9 rats) or anti-ComE1 antiserum (ComE1; cohort of 10 rats). Using Fisher’s exact test to compare percentages of bacteremic pups *P* = 0.0031 for PBS vs ComE1 and *P* = 0.0698 for PIS vs ComE1. Panel B) Bacteremic titers in infant rats pretreated with anti-ComE1 antisera 24 hours after infection. Filled dots represent the bacteremic titer in each individual animal in a cohort. The unfilled dot represents the average bacteremic titers in all members of the cohort. Values of 1 or below represent animals with no detectable bacteremia. Using the Kruskal-Wallis test to compare bacteremic titers (mean ±SD) *P* = 0.07 for PBS vs PIS, *P* = 0.0002 for PBS vs ComE1 and *P* = 0.01 for PIS vs ComE1. Panel C) Percentage of infected rats pre-treated with anti-Hel1 antisera with detectable bacteremia 24 hours after infection. Twenty-four hours prior to infection cohorts of infant rats were pretreated with phosphate-buffered saline (PBS; cohort of 10 rats), pre-immune serum (PIS; cohort of 9 rats) or anti-Hel1 antiserum (Hel1; cohort of 10 rats). Using Fisher’s exact test to compare percentages of bacteremic pups *P* = 0.0325 for both PBS vs Hel1 and PIS vs Hel1. Panel D) Bacteremic titers in infant rats pretreated with anti-Hel1 antiserum with detectable bacteremia 24 hours after infection. Filled dots represent the bacteremic titer in each individual animal in a cohort. The unfilled dot represents the average bacteremic titers in all members of the cohort. Values of 1 or below represent animals with no detectable bacteremia. Using the Kruskal-Wallis test to compare bacteremic titers (mean ±SD) *P* = 0.15 for PBS vs PIS, *P* = 0.0003 for PBS vs Hel1 and *P* = 0.0005 for PIS vs Hel1.

Each of these five epitopes was used in our immunization protocol. The TdeA1 peptide did not induce an antibody titer sufficient to proceed with further study of that antigen. Antisera raised to the OmpU1 peptide did not provide a significant protective effect in the infant rat model. Five of 9 infant rats in the OmpU1-antisera treated cohort and 9 of 10 infant rats in the control cohort had detectable bacteremia (*P* = 0.14). Seven of 10 infant rats pretreated with antiserum raised to ComE1 failed to develop bacteremia (Fig 4A). While the rate of bacteremia of the anti-ComE1 treated group was significantly lower than the rate for the PBS treated group (*P* = 0.0031) it did not significantly differ from the pre-immune serum treated group (*P* = 0.0698) probably to a small cohort size in the latter group (Fig 4A). However, the bacteremic titer in the anti-ComE1 antiserum cohort was significantly lower that seen in either of the control groups (Fig 4B). Antiserum raised to the Hel1 was significantly protective when given to infant rats 24 hours prior to challenge with NTHi strain R2866. While all rats pretreated with either PBS or the pre-immune serum had detectable bacteremia 24 hours after infection 5 of 10 animals pretreated with anti-Hel1 antiserum were abacteremic (*P* = 0.0325) (Fig 4C). Bacteremic titers were also significantly lower in those rats pretreated with anti-Hel1 antiserum than those rats pretreated with either PBS or pre-immune serum (Fig 4D). Antiserum raised to the Hel2 peptide gave similar results to those seen for Hel1 (data not shown).

## Discussion

There currently are no vaccines licensed for prevention of infection caused by NTHi. Although several potential vaccine candidates against NTHi have been evaluated. robust cross protection against NTHi strains has not been observed. For example the *H*. *influenzae* protein D component of the pneumococcal vaccine has demonstrated a 35% protection rate in a clinical trial [25]. From our studies protein D (encoded by *glpQ*) exhibits multiple variant residues among NTHi strains. This may account for the low protection rate. Alternatively, expression of protein D may vary among different NTHi strains. Thus, failure of previous vaccine candidates may arise in part from problems of target protein conservation and/or biological accessibility. In our study we sought to obviate the problem of lack of conservation. An initial step in this study was to identify the conserved core SEPs shared by all the NTHi. Most NTHi strains possessed approximately 90 genes encoding SEPs. Of these, several are either distinct to a particular isolate or restricted to a few isolates, and are thus unsuited as vaccine candidates. For example, the Hmw1A, Hmw1B, Hmw2A, Hmw2B, HgpA, HgpD and HgpE proteins are common among the NTHi, but not conserved in all [53,54]. Clearly, a large set of genomic sequences is required to exclude common, yet non-conserved proteins. From the 21 genomically sequenced, diverse NTHi isolates we have narrowed the core set of SEPs to 56 proteins. As more genomic sequences become available it is possible that this number may decrease further. However, based on our selection of strains previously characterized to represent the genetic breadth of the species, the number of genomes sequenced, and the conservation of core genes predicted by computer based models [55], the chance of elimination of a gene with a further increase in the number of sequenced genomes is possible but unlikely. Thus, we propose that 56 genes encode the core SEPs of the NTHi. The conservation across the species of these core SEPs suggests they are required for the survival and/or cellular fitness of the NTHi. Thus, finding conserved regions of these proteins was not surprising and probably represents their essential functionality for NTHi survival. Of these 56 core SEPs the structures of 41 have been modeled.

Initially, putative externally exposed loops were selected from the modeled SEPs based on the length of the conserved region. Regions containing greater than 10 amino acids were selected as possible linear epitopes. Over 80 such regions that showed complete identity were identified (S2 Table). The presence of conserved external loops suggests that these regions play a critical role in protein function. Alternatively, variations in these regions may be unnecessary if the regions are not available to the human immune system.

Almost 200 peptides from the core SEPs satisfied our initial screening criteria. While computer-assisted modeling and bioinformatics may identify putative exposed regions, antibody accessibility *in vivo* must be confirmed empirically. A number of confounding factors may render a putative epitope inaccessible. Gene regulation, spatial orientation of the loop itself, shielding by other loops and steric hindrance can render potential targets antibody inaccessible. To empirically determine *in vivo* antibody accessibility, an animal model was utilized. Of the 200 candidate peptides, 10 have been analyzed to determine their biological accessibility (Table 1).

Initially, the 5 peptides targeting regions of HxuC were analyzed. Peptides HxuC1 and HxuC2 generated antisera that were protective while HxuC3, HxuC4 and HxuC5 did not. Since these experiments were performed, new sequencing data have revealed that HxuC5 has two variant residues in the middle of the loop. In two of the newer sequences an isoleucine residue is substituted for a methionine residue, and in a third sequence a lysine is substituted for an arginine. The protein in strain R2866 has both the methionine and arginine residues. Thus, sequence heterogeneity cannot explain the lack of protection observed by antisera raised to peptide HxuC5.

Based on the availability of genomic sequences at the time of these studies, all of the peptides (with the exception of ComE1, Hel2 and TdeA) were designed to loops that were absolutely conserved across the NTHi. Peptides ComE1, Hel2 and TdeA were designed to match the inherent variability of the corresponding OM loop. Each had a single variant residue. To address this heterogeneity, two peptides were made for each sequence and an equimolar mixture of each was used to inoculate the adult rats. The outer membrane loop that the ComE1 peptide was designed from was estimated at 33 amino residues. From this a 20 residue region was selected based on maximal immunogenicity predicted by the AbDesigner algorithm [41]. Similarly the 20-mer peptide Hel1 was selected from an estimated exposed loop of 27 residues. Antisera raised to both of the Hel peptides and the ComE1 peptide were protective. Analysis of the specific immunogenicity of each peptide was beyond the scope of the present study.

It is unknown why only certain peptides stimulate protective antisera. Of the five synthetic peptide antigens targeting the HxuC protein, only two provided significant protection. Since the structure of the gated porins is well defined and the target appears surface exposed, steric hindrance may interfere with antibody accessibility to the non-protective loops. The same may also be true for antisera raised to the OmpU1 peptide since OmpU has a known β-barrel structure. Unlike HxuC, ComE and Hel are both amorphous structures with a suspected membrane anchor. *In situ*, the majority of the protein would be surface exposed. Selection of peptides from these sequences was based primarily on regions of predicted α-helices. While each peptide was modeled to be surface exposed, the clear protection against NTHi challenge in the infant rat provides empirical proof of surface exposure.

In summary, NTHi is an important public health burden without an obvious vaccine target. We hypothesize that likely successful targets may be regions of proteins that are both conserved and antibody available *in vivo*. Screening of well-characterized NTHi genome sequences identified 56 core SEPs present in all strains. These proteins were modeled to identify regions which may be available on the outer surface. Antisera raised to 10 externally exposed, conserved peptides were tested for their *in vivo* passive protective capacity. Of the 10 peptides, 5 induced sera that provided protection. These data provide proof of principle that certain conserved peptides may be antibody available and may be useful components of a vaccine. Additionally, these data provide a foundation to identify many other peptide targets for antibody production to protect against NTHi infection.

## Supporting Information

S1 Table Potential surface-exposed, highly conserved peptides of the NTHi. (XLSX)

S2 Table External peptides with 100% conservation. (XLSX)
